# The Effects of High‐ and Moderate‐Intensity Exercise on Epithelial Integrity Markers and Inflammatory Responses in Healthy Young Men

**DOI:** 10.1002/ejsc.70227

**Published:** 2026-07-23

**Authors:** Saeid Nikoukheslat, Hadi Pourmanaf, Vahid Sari‐Sarraf, Ramin Amirsasan, Dean E. Mills

**Affiliations:** ^1^ Faculty of Physical Education and Sport Sciences University of Tabriz Tabriz Iran; ^2^ School of Health and Medical Sciences University of Southern Queensland Ipswich Queensland Australia; ^3^ Respiratory and Exercise Physiology Research Group School of Health and Medical Sciences University of Southern Queensland Ipswich Queensland Australia; ^4^ Centre for Health Research Institute for Resilient Regions University of Southern Queensland Ipswich Queensland Australia

**Keywords:** CC16, CC16/SP‐D ratio, CRP, endurance exercise intensity, SP‐D

## Abstract

The effects of exercise intensity on airway epithelial integrity markers and inflammatory responses remain unknown. Therefore, we investigated the effects of high‐ and moderate‐intensity exercise on airway epithelial integrity markers (club cell protein 16 [CC16], surfactant protein D [SP‐D], and the CC16/SP‐D ratio), along with systemic inflammation assessed via high‐sensitivity C‐reactive protein [hs‐CRP] in healthy young men. Twenty healthy young men were randomly divided into two groups: high‐intensity (*n* = 10) and moderate‐intensity (*n* = 10) and both groups completed 20‐min of continuous intensity treadmill exercise. The exercise intensities for the high‐intensity and moderate‐intensity groups were 85%–90% and 65%–70% of maximum heart rate, respectively. Serum was collected before and at +1 and + 24 h after exercise. CC16, SP‐D, and hs‐CRP concentrations were measured using enzyme‐linked immunosorbent assay. A two‐way repeated measures analysis of variance suggested that CC16, SP‐D, CC16/SP‐D ratio, and hs‐CRP changed significantly over time (*p* < 0.05). Time × group interaction effects were observed for CC16 (*p* = 0.032) and CC16/SP‐D ratio (*p* = 0.010) but not for SP‐D (*p* = 0.824) and hs‐CRP (*p* = 0.422) concentrations. Post hoc analysis revealed that CC16 concentrations at +1 h (*p* = 0.002) and the CC16/SP‐D ratio at +24 h (*p* = 0.005) were higher for the high‐ compared to the moderate‐intensity group. Our findings suggest for the first time that exercise intensity has a significant impact on CC16 and the CC16/SP‐D ratio but does not affect the release of SP‐D or hs‐CRP.

## Highlights

- The effects of exercise intensity on airway epithelial integrity markers and inflammatory responses remain unknown.
- We investigated the effects of high‐ and moderate‐intensity exercise on airway epithelial integrity markers (club cell protein 16 [CC16], surfactant protein D [SP‐D], and the CC16/SP‐D ratio), along with systemic inflammation assessed via high‐sensitivity C‐reactive protein [hs‐CRP] in healthy young men.
- CC16, SP‐D, CC16/SP‐D ratio, and hs‐CRP concentrations changed significantly over time and the increase in CC16 (+1 h) and the CC16/SP‐D ratio (+24 h) were greater for the high‐ compared to the moderate‐intensity group, whereas there were no differences between the groups for SP‐D or hs‐CRP.
- Our findings suggest for the first time that exercise intensity has a significant impact on the CC16 and the CC16/SP‐D ratio but does not affect the release of SP‐D or hs‐CRP.

## 1 Introduction

Asthma is a complex multifactorial condition that is characterized by a range of symptoms including chronic airway inflammation, expiratory airflow limitation, and cough (Porsbjerg et al. 2023). The global incidence of asthma is increasing, and it affects an estimated 339 million individuals worldwide (Yuan et al. 2025). Exercise‐induced bronchoconstriction (EIB) is a form of asthma defined by the transient narrowing of the airways in response to physical exercise without other asthma signs (Pigakis et al. 2022). The prevalence of EIB among the general population is 5%–20% (Aggarwal et al. 2018; Rodriguez Bauza and Silveyra 2021), but the prevalence among athletes, particularly those engaged in high‐intensity aerobic exercise, has been shown to be greater at between 7% and 70% (Thirión‐Romero et al. 2025).

The underlying cause of EIB remains to be fully elucidated. However, the pathogenesis of the condition may be attributed to various factors, including airway epithelial injury, airway inflammation, and oxidative stress (Stenfors et al. 2022). During high‐intensity aerobic exercise, endurance athletes can increase their ventilation by a factor of 10–20 in comparison to rest (Boulet and O'Byrne 2015). However, this heightened ventilation could lead to a deterioration in the desired humidity and temperature of the inspired air, resulting in water evaporation from the airway surface (Karamaoun et al. 2022; Pigakis et al. 2022; Price et al. 2013). Accelerated evaporation causes dehydration of the airway surface liquid, which increases shear stress and promotes epithelial cell detachment or sloughing (Kippelen and Anderson 2012). Subsequently, epithelial integrity markers, including club cell protein 16 (CC16) and alveolar surfactant‐associated serum proteins (SP‐A, SP‐B, and SP‐D), are released into the bloodstream (Combes et al. 2019; Karamaoun et al. 2022; Pourmanaf et al. 2020).

CC16 is a protein with a molecular weight of 10–16 kDa that is primarily synthesized by nonciliated bronchial epithelial cells in the airway epithelium. This protein has been identified as a reliable marker of airway epithelial barrier integrity and has been demonstrated to possess anti‐inflammatory properties (Guo et al. 2025; Heltborg et al. 2024; Voraphani et al. 2023). SP‐D, a collagen glycoprotein, is a soluble molecule of the innate immune system that plays a critical role as an anti‐infective and immunomodulatory agent (Guo et al. 2025; Heltborg et al. 2024; Tiezzi et al. 2021; Watson et al. 2020). The serum CC16/SP‐D ratio is also a specific and sensitive test to detect lung epithelium damage caused by toxicants (Bernard 2008; Bernard et al. 2007; Robin et al. 2002). Numerous studies have reported that acute aerobic exercise increases serum CC16 concentrations following exercise (L. Eklund et al. 2021; L. M. Eklund et al. 2022; Pourmanaf et al. 2023; Stenfors et al. 2022; Tufvesson et al. 2013). However, the results concerning SP‐D (Combes et al. 2019; Pourmanaf et al. 2020; Pourmanaf et al. 2023) and the CC16/SP‐D ratio (Combes et al. 2019; Pourmanaf et al. 2020; Pourmanaf et al. 2023) are inconclusive and limited. Moreover, recent findings have reported that increased ventilation during exercise can result in airway inflammation (Karamaoun et al. 2022). The serum concentration of high sensitivity C‐reactive protein (hs‐CRP) is widely recognized as a biomarker of systemic inflammation. However, it may also provide complementary information regarding the inflammatory response of the respiratory system associated with physiological stress (Shimoda et al. 2015), with the potential to increase following acute aerobic exercise (Mendham et al. 2011).

Although prior studies have confirmed the release of CC16 following acute endurance exercise, no study has examined the effect of endurance exercise intensity. Moreover, the current literature has yielded limited studies that have investigated the effect of aerobic exercise on SP‐D, the CC16/SP‐D ratio, and hs‐CRP, resulting in an absence of conclusive evidence regarding these markers. Therefore, we investigated the effects of high‐ and moderate‐intensity exercise on airway epithelial integrity markers (CC16, SP‐D, and CC16/SP‐D ratio) as the primary focus, with systemic inflammation (hs‐CRP) assessed as a secondary outcome, in healthy young men. We hypothesized that higher‐intensity endurance exercise would lead to greater airway epithelial damage, accompanied by a secondary systemic inflammatory response.

## 2 Methods

### 2.1 Participants

Twenty healthy adult males aged 18–30 years, who participated in recreational physical activity 1 to 3 times per week (including badminton, volleyball, basketball, and football), volunteered to participate in the study. The inclusion criteria were as follows: (1) participants with a body mass index (BMI) between 18 and 25 kg/m^2^; and no prior history of (2) pulmonary and cardiovascular diseases; (3) tobacco smoking for at least 6 months prior to the study; and (4) using *β*_2_‐agonists or any anti‐inflammatory medications or supplements in the past month. A customized questionnaire was used to collect data regarding personal and family history of diseases, dietary habits, physical activity levels, and the history of tobacco smoking. Prior to participation, all individuals were informed verbally and in writing about the objectives of the study, and written consent was obtained from each participant. The study was approved by the local Research Ethics Committee, which adheres to the Declaration of Helsinki. The protocol of the study was also registered.

### 2.2 Experimental Design

Participants were randomly divided into two groups: high‐intensity (*n* = 10) and moderate‐intensity (*n* = 10) exercise. The randomization process was performed on an individual basis, employing the randomizer software (RandList 1.2.www.randomisation.net). Opaque and sealed envelopes were used to ensure the allocation concealment process remained secure. The groups were encoded, and the codes were sealed by an independent investigator. Participants were required to visit the Exercise Physiology Laboratory for two visits. During the first visit, anthropometric characteristics were initially measured. Height was measured using a wall‐mounted stadiometer (Seca 206, Seca, Hamburg, Germany). Body mass, body fat percentage, and muscle mass were assessed via bioelectrical impedance analysis (720 Body Composition Analyzer; Inbody Co. Ltd.ˏ Seoulˏ Korea), with participants standing barefoot on the device, wearing minimal clothing, and holding the device's handles. Subsequently, participants' maximum oxygen consumption ($\dot{V}$O_2_max) was estimated using the Cooper test (K. H. Cooper 1968) in which participants ran as far as possible for 12 minutes on a standard racetrack and $\dot{V}$O_2_max was predicted from the distance covered using the following formula: $\dot{V}$O_2_max (mL/kg/min) = (22.351 × distance covered in kilometres) − 11.288.

One week after the first visit, the high‐intensity and moderate‐intensity groups engaged in a 20‐min session of continuous endurance exercise on a treadmill (Run Excite 700; Technogym, Cesena, Italy) within the time window of 8:00 a.m. to 12:00 p.m. Participants completed a 10‐min warm‐up prior to beginning the exercise including 5 minutes of light running followed by 5 minutes of stretching exercises involving the major muscle groups. Subsequently, participants undertook the treadmill exercise. The initial treadmill speeds were set at 9 and 7.5 km/h for the high‐ and moderate‐intensity groups, respectively. These speeds varied throughout the exercise based on the intensity assigned to each individual. The incline of the treadmill was set to 0°. The exercise intensity for the high‐ and moderate‐intensity groups was set at 65%–70% and 85%–90% of maximum heart rate (MHR), respectively. These exercise intensities were based upon the American College of Sports Medicine classifications for moderate (64%–76% of MHR) and vigorous (77%–95% of MHR) intensity exercise (ACSM 2020; Bishop et al. 2025). The Tanaka equation (MHR = 208 − (0.7 × Age)) was used to estimate MHR (Tanaka et al. 2001). Heart rate was measured continuously during exercise using short‐range telemetry (Polar T31 C, Polar, Kempele, Finland). The temperature and humidity of the laboratory were maintained within the range of 23°C–24°C and 50%–60%, respectively. At the end of the exercise session, participants were instructed to perform 10 minutes of cool‐down exercises. Participants arrived at the laboratory 4 h postprandially having abstained from caffeine in the 4 h before testing and were informed to refrain from participating in any type of physical activity for 48 h before testing.

### 2.3 Blood Sampling and Enzyme‐Linked Immunosorbent Assays

Blood samples (5 mL) were collected from the antecubital vein of participants using sterile needles and serum tubes (Hebei Xinle Sci & Tech Co., China) at three time points: before (baseline) and +1 h and +24 h after treadmill exercise. We based these time points on previous research. However, changes in these markers at longer time points (e.g., one, two, or 3 days after exercise) have not yet been investigated. Therefore, we selected +24 h after exercise as an additional sampling time point. These samples were used to measure serum concentrations of CC16, SP‐D, and hs‐CRP. Samples were permitted to undergo clotting at room temperature for approximately 30–60 minutes prior to centrifugation at 3000 rpm for 10 min. Subsequently, serum was distributed into 1.8‐mL aliquots and stored at a temperature of −20°C until biochemical assays were performed. Serum concentrations of CC16, SP‐D, and hs‐CRP were measured using enzyme‐linked immunosorbent assays (ELISA) (ZellBio GmbH, Veltinerweg, Germany), utilizing a biotin double antibody sandwich technology. The assay ranges for CC16 (Cat. No: ZB‐10073C‐H9648), SP‐D (Cat. No: ZB‐11072‐C‐H9648), and hs‐CRP (Cat. No: ZB‐11805S‐H9648) were 0.5–16 ng/mL, 1–32 ng/mL, and 156–10000 ng/mL, respectively. ELISAs were performed by the following instructions for each specific kit in duplicate. In order to minimize the effect of interassay variation, the markers from both groups were measured using the same assay plate.

### 2.4 Statistical Analysis

The G*Power software (version 3.1.9.2) was used to determine the necessary sample size, based on CC16 concentrations (Effect size = 0.5, *α* = 0.05, Power = 0.8). Statistical analyses were performed using SPSS software, version 26 (IBM, Chicago, IL, USA). The Shapiro–Wilk test was employed to evaluate the normality of the variables which were normally distributed. Between‐group comparisons including participant characteristics and treadmill exercise were made using independent *t*‐tests. A repeated measure analysis of variance (ANOVA) was used to determine the effects of time (baseline, +1 h and + 24 h) and group (high‐ vs. moderate intensity) on the CC16, SP‐D, CC16/SP‐D ratio and hs‐CRP responses to exercise. Following significant main effects, the Bonferroni post hoc test was performed to compare the time points of +1 h and +24 h after exercise with baseline values and between the high‐ and moderate‐intensity exercise groups. Results are presented as means ± standard deviation. Statistical significance was set at *p* < 0.05.

## 3 Results

### 3.1 Participants Characteristics and Treadmill Exercise

**FIGURE 1:**
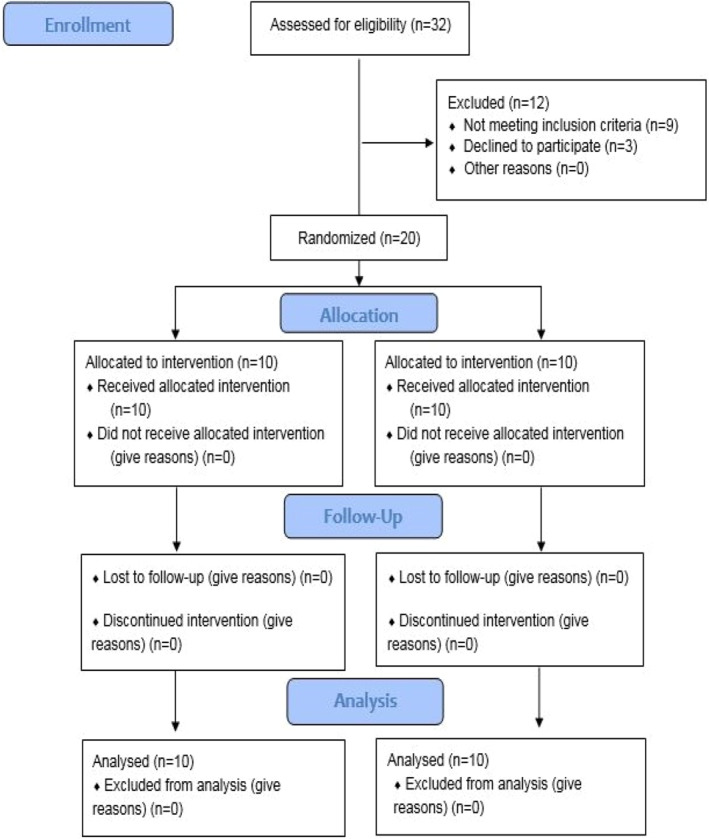
CONSORT flow diagram.

A total of 20 participants completed the study (Figure 1; Consolidated Standards of Reporting Trials (CONSORT) diagram). The anthropometric characteristics, estimated $\dot{V}$O_2_max, exercise distance, exercise speed, and mean heart rate during the treadmill exercise are provided in Table 1. There were no differences between the groups for the anthropometric characteristics (*p* > 0.05) and estimated $\dot{V}$O_2_max. Exercise distance and speed and mean heart rate during the treadmill exercise were higher for the high‐ compared to the moderate‐intensity group (*p* < 0.05).

**TABLE 1:** Anthropometric characteristics, physical activity, estimated heart rate and maximal oxygen uptake ($\dot{V}$O_2_max), and treadmill exercise data for the high‐ and moderate‐intensity groups.

Variables	Moderate‐intensity (*n* = 10)	High‐intensity (*n* = 10)	*p* value
Age (years)	21 ± 3	22 ± 2	0.569
Height (m)	1.80 ± 0.07	1.82 ± 0.05	0.657
Body mass (kg)	74 ± 7	77 ± 8	0.455
Body mass index (kg/m^2^)	22.82 ± 2.06	23.16 ± 1.67	0.654
Body fat (%)	16 ± 5	15 ± 4	0.458
Body muscle (kg)	36 ± 3	37 ± 4	0.398
Physical activity (min/week)	179 ± 111	253 ± 79	0.103
Estimated maximal heart rate (beats/min)	194 ± 2	193 ± 1	0.571
Distance covered during cooper test (km)	2.40 ± 0.37	2.33 ± 0.27	0.835
Estimated $\dot{V}$O_2_max (mL/kg/min)	42.40 ± 8.42	41.61 ± 6.23	0.809
Exercise distance (km)	2.16 ± 0.32	3.30 ± 0.35	**0.001**
Exercise speed (km/h)	6.46 ± 0.97	9.90 ± 1.04	**0.001**
Mean heart rate during exercise (beats/min)	137 ± 7	173 ± 5	**0.001**

*Note:* Data are presented as mean ± SD. Bold *p* values denote significant (*p* < 0.05) differences between groups.

### 3.2 Club Cell Protein 16

**FIGURE 2:**
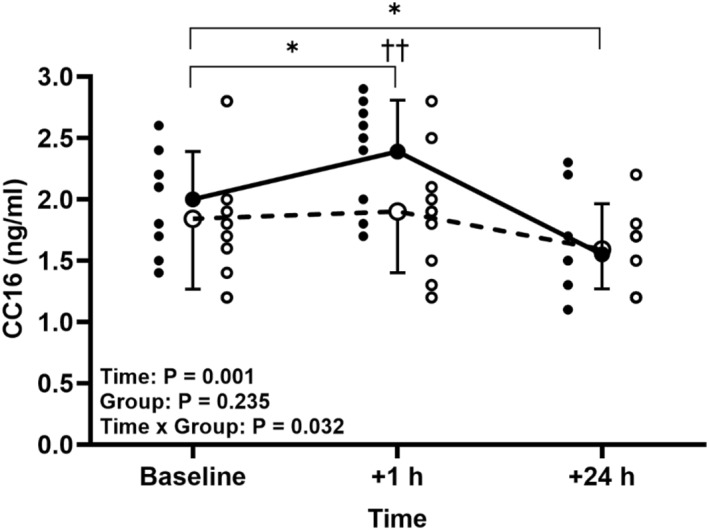
Serum club cell 16 (CC16) concentrations at baseline, and at +1 h and +24 h after exercise for the high‐ (filled circles) and moderate‐intensity (open circles) groups. Individual circles represent individual participants. The smaller number of circles at certain times is due to the same data number. Mean values are shown with connecting lines and error bars represent standard deviation. Significantly different from baseline for the high‐intensity group (*p < 0.01). Significantly different between the high‐ and moderate‐intensity groups (^††^ *p* < 0.01).

The responses of CC16 to exercise for the high‐ and moderate‐intensity groups are shown in Figure 2. The repeated measures ANOVA revealed that serum CC16 concentrations changed significantly over time (*p* = 0.001). The post hoc analysis revealed that compared to baseline concentrations, CC16 significantly increased at +1 h (*p* = 0.024) and decreased at +24 h (*p* = 0.014) in the high‐intensity group, whereas no significant differences were identified in the moderate‐intensity group at +1 h (*p* = 0.95) or +24 h (*p* = 0.283). There were no main effects of group (*p* = 0.235), but there was a time × group interaction (*p* = 0.032). The post hoc analysis revealed that CC16 was higher at +1 h (*p* = 0.002), but not different at +24 h (*p* = 1.000) for the high‐ compared to the moderate‐intensity group.

### 3.3 Surfactant Protein D

**FIGURE 3:**
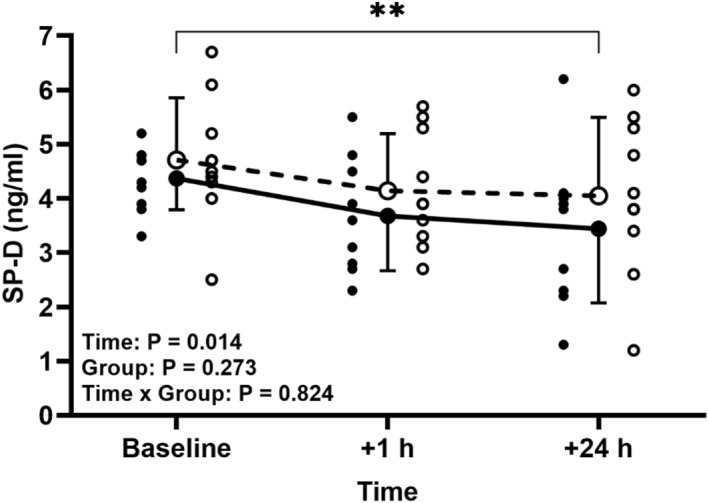
Surfactant protein D (SP‐D) concentrations at baseline, and +1 h and +24 h after exercise for the high‐ (filled circles) and moderate‐intensity (open circles) groups. Individual circles represent individual participants. The smaller number of circles at certain times is due to the same data number. Mean values are shown with connecting lines and error bars represent standard deviation. Significantly different from baseline for the high‐intensity group (**p < 0.01).

The responses of SP‐D to exercise for the high‐ and moderate‐intensity groups are shown in Figure 3. The repeated measures ANOVA revealed that serum SP‐D concentrations changed significantly with time (*p* = 0.014). The post hoc analyses revealed no pairwise differences between baseline concentrations and +1 h for the high‐intensity (*p* = 0.193) and moderate‐intensity (*p* = 0.061) groups. However, a significant decrease was observed at +24 h after exercise compared to baseline for the high‐intensity group (*p* = 0.001), but not for the moderate‐intensity group (*p* = 0.176). There were no main effects of group (*p* = 0.273) or time × group interaction effects (*p* = 0.824).

### 3.4 Club Cell 16 (CC16)/Surfactant Protein D (SP‐D) Ratio

**FIGURE 4:**
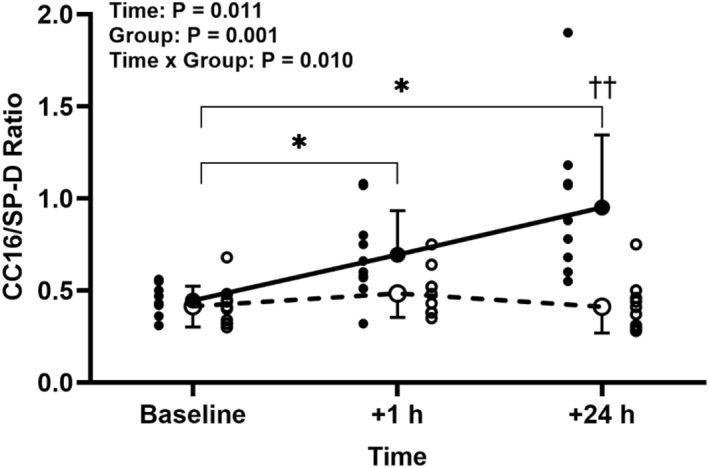
Club cell 16 (CC16)/surfactant protein D (SP‐D) ratio at baseline, and +1 h and +24 h after exercise for the high‐ (filled circles) and moderate‐intensity (open circles) groups. Individual circles represent individual participants. The smaller number of circles at certain times is due to the same data number. Mean values are shown with connecting lines and error bars represent standard deviation. Significantly different from baseline for the high‐intensity group (*p < 0.01). Significantly different between the high‐ and moderate‐intensity groups (^††^ *p* < 0.01).

The responses of the CC16/SP‐D ratio to exercise for the high‐ and moderate‐intensity groups are shown in Figure 4. The repeated measures ANOVA revealed that the CC16/SP‐D ratio changed significantly with time (*p* = 0.011). The post hoc analysis revealed that compared to baseline concentrations, the CC16/SP‐D ratio increased significantly at +1 h (*p* = 0.018) and +24 h (*p* = 0.014) in the high‐intensity group. However, in the moderate‐intensity group, no changes were observed at +1 h (*p* = 0.363) or +24 h (*p* = 0.950). There were main effects of group (*p* = 0.001) and time × group interaction (*p* = 0.010). The post hoc analysis revealed that the CC16/SP‐D ratio was not different at +1 h (*p* = 0.085) but was higher at +24 h (*p* = 0.005) for the high‐ compared to the moderate‐intensity group.

### 3.5 C‐Reactive Protein

**FIGURE 5:**
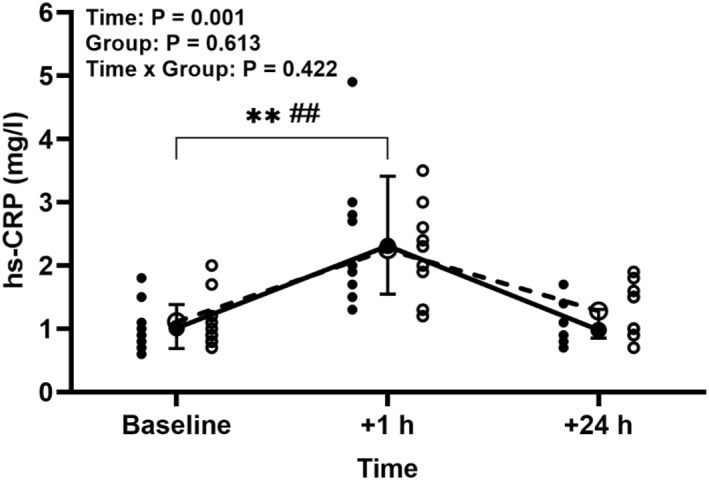
High sensitivity C‐reactive protein (hs‐CRP) concentrations at baseline, and +1 h and +24 h after exercise for the high‐ (filled circles) and moderate‐intensity (open circles) groups. Individual circles represent individual participants. The smaller number of circles at certain times is due to the same data number. Mean values are shown with connecting lines and error bars represent standard deviation. Significantly different from baseline for the high‐ (**p < 0.01) and moderate‐intensity (^##^ *p* < 0.01) groups.

The responses of hs‐CRP to exercise for the high‐ and moderate‐intensity groups are shown in Figure 5. The repeated measures ANOVA revealed that hs‐CRP concentration changed significantly over time (*p* = 0.001). The post hoc analysis revealed that compared to baseline concentrations, hs‐CRP increased significantly at +1 h for the high‐ (*p* = 0.005) and moderate‐intensity groups (*p* = 0.001) and returned to baseline concentrations at +24 h for both the high‐ (*p* = 0.950) and moderate‐intensity (*p* = 0.687) groups. There were no main effects of group (*p* = 0.613) or time × group interaction (*p* = 0.422).

## 4 Discussion

### 4.1 Main Findings

We investigated the effects of high‐ and moderate‐intensity exercise on epithelial integrity (CC16, SP‐D, and CC16/SP‐D ratio) and systemic inflammatory (hs‐CRP) markers in healthy young men. Our main findings are two‐fold. Firstly, CC16, SP‐D, CC16/SP‐D ratio, and hs‐CRP concentrations changed significantly over time. CC16 (+1 h) and the CC16/SP‐D ratio (+1 and + 24 h) increased following exercise for the high‐intensity group only, whereas hs‐CRP increased following exercise in both groups. SP‐D decreased at +24 h for the high‐intensity group only. Secondly, the increase in CC16 (+1 h) and the CC16/SP‐D ratio (+24 h) were greater for the high‐ compared to the moderate‐intensity group, whereas there were no differences between the groups for SP‐D or hs‐CRP. Our findings suggest for the first time that intensity has a significant impact on CC16 and the CC16/SP‐D ratio but does not affect the release of SP‐D or hs‐CRP.

### 4.2 Effect of Exercise on Epithelial Integrity Markers

CC16, SP‐D and the CC16/SP‐D ratio have been identified as reliable markers of airway epithelial integrity (Bernard 2008). An increase in these markers in the blood indicates the presence of respiratory disease or airway damage (Combes et al. 2019; Nikniaz et al. 2021). Increased ventilation during exercise leads to water evaporation and subsequently dehydration of the airway surface liquid. This, in turn, increases shear stress and transmural pressure gradients across the airway epithelium, leading to damage and release of specific markers of epithelial integrity (Combes et al. 2019; Pigakis et al. 2022; Price et al. 2013). We observed an increase in CC16 at +1 h and the CC16/SP‐D ratio at +1 and + 24 h following exercise for the high‐intensity group only. We also observed a decrease in SP‐D at +24 h for the high‐intensity group only. Nevertheless, additional intervals within and following the 24‐h period would provide a more comprehensive understanding of the changes in these biomarkers in response to exercise.

Our findings support others that have reported an increase in serum CC16 concentrations immediately following acute endurance exercise (Broeckaert et al. 2000; L. Eklund et al. 2021; L. M. Eklund et al. 2022; Nanson et al. 2001; Stenfors et al. 2022; Tufvesson et al. 2013). However, research on the acute effects of endurance exercise on SP‐D and the CC16/SP‐D ratio is limited, and the results of these studies are also contradictory (Combes et al. 2019; Font‐Ribera et al. 2010; Pourmanaf et al. 2020). In a recent systematic review and meta‐analysis, we investigated the effects of acute endurance exercise on CC16, SP‐D, and CC16/SP‐D ratio in both athletic and nonathletic adults (Pourmanaf et al. 2023). The results of the meta‐analysis demonstrated that immediately following endurance exercise, CC16 and the CC16/SP‐D ratio increased. However, no significant changes in serum SP‐D concentrations were observed. The mechanisms for the greater increase in CC16 and the CC16/SP‐D ratio following high‐intensity exercise probably relate to shear stress and transmural pressure gradients across the airway epithelium, which result from increased ventilation (Pigakis et al. 2022; Price et al. 2013). It is likely that a longer bout of exercise performed at the same exercise intensities reported here may also result in a greater response of these airway epithelial integrity markers. Further, outdoor exercise such as marathon running with a greater minute ventilation than the present study and undertaken in high air pollutant concentrations may further exacerbate airway health risks.

Exposure to air pollutants during endurance exercise in outdoor environments may exacerbate respiratory stress, as increased ventilation enhances the inhaled dose and deposition of particulate matter in the airways, potentially leading to greater airway inflammation and epithelial injury in endurance athletes. Given that particulate matter can induce oxidative stress, airway inflammation, and epithelial injury—effects that may be exacerbated during exercise due to increased ventilation—future studies should consider monitoring or controlling indoor air quality to further isolate the effects of exercise intensity on airway epithelial outcomes (Wei et al. 2024). It should be noted that we did not directly assess environmental air quality parameters, including particulate matter concentrations (PM2.5, PM5, and PM10), during the experimental visits. Although all exercise trials in the present study were conducted in a controlled indoor laboratory setting to minimize environmental variability, the absence of direct air pollution measurements prevents complete exclusion of potential residual exposure effects.

We also observed that at +24 h, CC16 and SP‐D were reduced below baseline concentrations in the high‐intensity group only. It is our understanding, based on the available evidence, that this is the first study to assess the serum concentration of CC16 and SP‐D 24 hours after endurance exercise. Therefore, the mechanism of CC16 and SP‐D reduction is not yet clear. However, this could relate to a decrease in the release of these markers from airway epithelial cells or their excretion in the urine within 24 hours of the exercise intervention.

### 4.3 Effect of Exercise on C‐Reactive Protein

hs‐CRP is a protein that has been confirmed as a sensitive biomarker of inflammation (J. Cooper et al. 2023; Rizo‐Téllez et al. 2023). Numerous studies have identified elevated baseline serum hs‐CRP concentrations in a variety of diseases, which have been proposed as a diagnostic tool to monitor disease progression (Rizo‐Téllez et al. 2023). Evidence has reported that elevated serum hs‐CRP concentrations are associated with specific markers of airway inflammation and impaired pulmonary function, suggesting that hs‐CRP may reflect systemic inflammatory responses that are related to airway inflammatory processes (Shimoda et al. 2015). Previous studies have demonstrated an increase in serum hs‐CRP concentrations following exercise (Lara Fernandes et al. 2011; Liakos et al. 2025; Mendham et al. 2011). In agreement with these findings, the present study suggested a significant increase in serum hs‐CRP concentrations following 20 minutes of continuous endurance exercise. However, there were no differences between the high‐ and moderate‐intensity groups in this response. This finding supports a systematic review that concluded hs‐CRP increased after both intense and moderate exercise (Cerqueira et al. 2019). It is likely that there are no differences between high‐ and moderate‐intensity exercise on the hs‐CRP response to exercise in healthy young men. The return of serum hs‐CRP concentrations to baseline values following exercise indicates that the increase in hs‐CRP is presumably transient, reverting to baseline after a 24‐h period.

Although hs‐CRP was included to reflect the systemic inflammatory response to exercise, it does not represent a specific marker of airway inflammation. Therefore, we may not have fully captured the complexity of exercise‐induced respiratory inflammatory processes. Future studies are encouraged to include additional airway‐specific inflammatory markers such as cytokines or exhaled nitric oxide to provide a more comprehensive evaluation of respiratory responses to exercise.

### 4.4 Limitations and Future Studies

Firstly, we only recruited males. However, the purpose of this study was to elucidate the effects of high‐ and moderate‐intensity exercise on airway inflammatory and epithelial integrity markers rather than to address potential sex‐based differences, which we recognize are present in respiratory physiology (Dominelli and Molgat‐Seon 2022). It could be hypothesized that the smaller airway size and greater work of breathing observed in women may lead to a greater release of inflammatory and epithelial integrity markers for any given ventilation. Therefore, future investigations should explore the impact of sex on changes in the release of inflammatory and epithelial integrity markers following exercise. Secondly, evidence from earlier studies points to an association between the ventilatory threshold and the airway epithelial dehydration threshold, which marks the onset of epithelial cell damage (Karamaoun et al. 2022). In the present study, minute ventilation was not controlled; therefore, it is recommended that this variable be accounted for in future research. Finally, the present study is lacking an objective physiological marker for determining exercise intensity, such as blood lactate concentration or oxygen uptake. The inclusion of these measurements could have provided a more accurate estimation of exercise intensity and improved the interpretation of the observed physiological responses. Therefore, future studies are recommended to incorporate physiological markers to allow for a more comprehensive evaluation of exercise intensity.

## 5 Conclusion

Our findings suggest for the first time that the epithelial integrity marker CC16 and the CC16/SP‐D ratio were greater for high‐ compared to moderate‐intensity exercise, whereas there were no differences between exercise types for SP‐D or the systemic inflammatory marker hs‐CRP. These findings suggest that airway epithelial injury may occur to a greater extent following high‐ compared to moderate‐intensity exercise and should be considered when designing aerobic exercise training programs or protecting the respiratory health of individuals undertaking repeated, vigorous exercise.

## Author Contributions

S.N., and H.P. designed the project and aided in interpreting the results. H.P. directed the project, developed the theoretical framework, and drafted the manuscript. S.N., H.P., and D.E.M. performed the analysis, designed the figures and interpreted the results. S.N., H.P., and D.E.M. wrote the manuscript. All authors edited the manuscript. All authors provided critical feedback and helped shape the research, analysis and manuscript. All authors read and approved the final manuscript.

## Funding

The authors have nothing to report.

## Consent

Informed consent was obtained from all individual participants included in the study. The authors affirm that human research participants provided informed consent for publication of their data.

## Conflicts of Interest

The authors declare no conflicts of interest.

## Data Availability

The datasets used and/or analyzed during the current study available from the corresponding author on reasonable request.
